# Treatment of Growth Hormone Deficiency via Daily Intravascular Injections in a Child with Bleeding Disorder

**DOI:** 10.1155/2021/7865398

**Published:** 2021-06-17

**Authors:** Emir Tas, Serife E. Uzun, Vildan Tas, Juan D. Mejia-Otero

**Affiliations:** ^1^Department of Pediatrics, University of Arkansas for Medical Sciences, Little Rock, AR, USA; ^2^College of Osteopathic Medicine, New York Institute of Technology, Glen Head, Long Island, NY, USA

## Abstract

**Objectives:**

The standard of treatment for pediatric growth hormone deficiency (GHD) is daily subcutaneous recombinant human growth hormone (rhGH) injections. The efficacy of rhGH treatment given as daily intravenous (IV) boluses is not known. *Case Presentation*. A female with protein C deficiency, a severe bleeding disorder characterized by thrombosis formation, was diagnosed with GHD at age four years. She has been receiving daily protein C infusion through a permanent port since the newborn period. GHD was treated with daily IV rhGH boluses given through the port following protein C infusion. She has reached a growth rate of 12 cm/year and had no side effects. Surprisingly, serum insulin-like growth factor-1 (IGF1) levels did not rise despite an excellent clinical response.

**Conclusions:**

IV administration may be an alternative route for GHD treatment in eligible patients with permanent vascular access. A rise in serum IGF1 levels may not be needed to achieve the growth-promoting effect of rhGH.

## 1. Introduction

Short stature is one of the most common referral reasons to pediatric endocrinology clinics. Among the various etiologies of short stature, growth hormone (GH) deficiency is the most common endocrine cause. Growth hormone deficiency (GHD) is estimated to have a prevalence of 1 in 4,000 to 10,000 and can be due to genetic defects, acquired causes, or idiopathic. Untreated GHD may have a profound impact on the overall health of the affected children with lasting consequences beyond childhood. Presenting symptoms and signs vary depending on the age of onset, but children with GHD usually come to medical attention with decelerated or stunted linear growth, delayed bone development, and delayed puberty. Screening tests for suspected GHD include measurement of serum growth factors, particularly insulin-like growth factor (IGF)-1, which is also used to assess adherence and monitor safety and as a guide to titrate GH dose during treatment. IGF1 is the primary mediator of GH-stimulated bone growth. Currently, the standard treatment of GHD involves daily subcutaneous injections of recombinant human growth hormone (rhGH). However, this administration route may be a source of stress in parents of children with severe underlying bleeding pathologies because of cutaneous bleeding risk. Although the likelihood of hitting a large vein in subcutaneous fat is practically zero, the parental anxiety associated with this may lead to nonadherence to GH treatment in such patients. There is a lack of knowledge regarding the efficacy and safety of daily intravenous (IV) administration of rhGH to treat GHD. We report the case of a patient with protein C deficiency who was diagnosed with GHD and successfully treated via daily IV rhGH boluses through an established port. To our knowledge, the IV route has not been previously used for long-term rhGH replacement therapy.

## 2. Case Presentation

A 2-year 5-month-old female was referred to for evaluation of short stature. She was born at term with a birth weight of 3845 grams and a length of 51 cm. Her history was notable for prolonged Neonatal Intensive-Care Unit (NICU) stay due to disseminated intravascular coagulation (DIC) characterized by multiple skin bruises and respiratory distress. She required a chest tube and G-tube placement to treat chylothorax and feeding problems, respectively, and transfusion of multiple blood products to manage DIC. After an extensive workup, she was diagnosed with protein C deficiency and has begun daily IV protein C concentrate (Ceprotin®) treatment. She had no pulmonary or cardiac comorbidities upon discharge from the NICU around four months of age. The brain magnetic resonance imaging (MRI) showed nonspecific white matter injury with multiple small subdural bleeds with no known neurological sequela. She also developed right-sided blindness due to retinal detachment.

**Figure 1:**
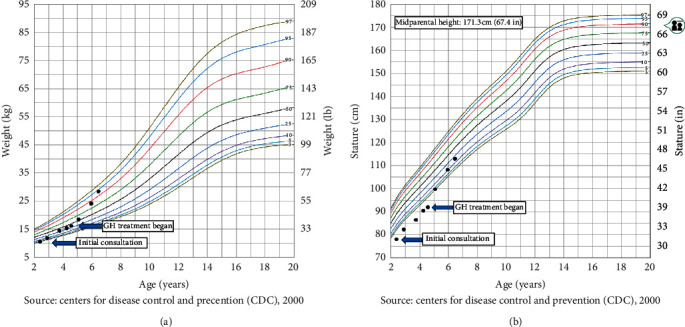
The weight (a) and height (b) data, at the time of initial consultation and when the growth hormone treatment has begun, indicated by arrows. GH, growth hormone.

On presentation to the endocrinology clinic, she plotted below one percentile in the height-for-age and at the third percentile in the weight-for-age charts (Figure 1).

She was in no acute distress and had normal vital signs. She was developmentally at par for age. Physical examination was notable for the presence of a subcutaneous port-a-cath on the left upper chest for daily protein C concentrate infusion and well-healed small scar tissues on her chest and abdomen. She had no red reflex on the right eye and self-amputated toes on the left foot. There were no organomegaly or any other dysmorphic features. Laboratory showed undetectable insulin-like growth factor-1 (IGF-1) (<15 ng/mL) and low IGF-binding protein-3 (BP-3) (0.65 mg/L, reference range 0.8–3.9) levels, but normal thyroid studies (Table 1).

Over the next two years, she did not show catch-up growth despite improved nutritional status (Figure 1). She failed to demonstrate a normal response to the arginine-clonidine provocation test with a peak GH level of only 4.4 ng/mL and was diagnosed with GHD. Repeat brain MRI showed hypoplastic anterior pituitary without any evidence of residual white matter damage. Recombinant hGH therapy was recommended. Due to concerns of extensive bruising with daily rhGH subcutaneous injections, parents requested a trial of IV administration of rhGH therapy via her port-a-cath. They agreed to switch to subcutaneous administration if there were poor response. Parents were made aware of the off-label use of IV rhGH therapy and signed informed consent after obtaining clearance from the hematologist. The patient started standard rhGH therapy at a standard subcutaneous dose (0.3 mg/kg/week) at age 4 years and 9 months via daily IV boluses through her port-a-cath following daily protein C infusions. She had an excellent response with a growth velocity of 12 cm/year in the first year of treatment without any adverse effects. Surveillance tests for thyroid and glucose tolerance were normal. Linear growth was accompanied by a modest increase in serum IGF-1 levels a few months after starting rhGH therapy with a slight further rise in serum IGF-1 levels after 16 months of rhGH therapy (Table 1).

## 3. Discussion

Herein, we describe a child with congenital protein C deficiency who was later diagnosed with GHD. Congenital protein C deficiency is a rare and severe bleeding disorder, characterized by a hypercoagulable state as a result of homozygous or compound heterozygous mutation of the PROC gene [1]. Affected infants may present with purpura fulminans, disseminated intravascular coagulation (DIC), or intracranial hemorrhage due to venous thrombosis [2]. Prompt recognition of this condition is important as a delay in treatment initiation may result in irreversible consequences. Our patient presented with DIC in the newborn period with extensive skin bruising and has then suffered blindness, self-amputated toes, and white matter changes in the brain. She has had no further bleeding after protein C treatment has begun. However, she was found to have a small anterior pituitary gland while being worked up for GHD. Schmitt et al. described a case with suspected protein C deficiency who later developed hypothalamic failure manifesting with combined pituitary hormone deficiencies, including GHD [3]. Cerebrovascular occlusion due to protein C deficiency was hypothesized to be the etiology of panhypopituitarism. To our knowledge, isolated GHD has not been described previously in a patient with protein C deficiency.

Growth hormone has multiple effects on various tissues and organs in the body. Besides playing a crucial role in linear growth, it also has vital functions in metabolism and cardiovascular physiology [4]. GH regulates the endothelial function and hepatic synthesis of some coagulation factors. Studies in adults linked GHD and adverse cardiovascular outcomes, including increased thromboembolic events, but pediatric data are lacking [5]. Therefore, diagnosis and treatment of GHD in a child with an underlying bleeding disorder maybe even more important than simply focusing on the effect of GH on linear growth.

The somatomedin hypothesis suggests that GH mediates its functions, including bone growth, mainly through the induction of hepatic IGF1, which activates transmembranous receptors in chondrocytes [6]. However, Wu et al., in their animal studies, demonstrated that GH could independently promote linear bone growth even when the local IGF1 action is prevented [7]. Although the serum IGF1 level of our patient has fairly increased following over one year of GH replacement, it has remained below two standard deviations for age and sex. Our observation supports the notion that GH may promote linear growth independent of a significant increase in serum IGF1 levels even in humans.

The current standard approach for the treatment of GHD is daily subcutaneous injection of recombinant rhGH. However, this route of administration presented a challenge in our patient due to the risk of extensive bruising. Per parent's request and after clearance from her hematologist, we agreed to attempt a trial of intravenous administration of rhGH via her port-a-cath with an option to switch to subcutaneous administration if clinical responses were suboptimal. The half-life of exogenous rhGH is estimated to be around 10–15 minutes [8]. Jørgensen et al. showed that a single IV rhGH bolus in healthy adult men increased mRNA expression of IGF1 in adipose, but not in muscle tissue [9]. In a previous study conducted by the same group, the researchers compared the effects of three different IV rhGH administration schedules (two large boluses at 2000 and 0200 hours. vs. eight small boluses separated at 3-hour intervals vs. continuous infusion between 2000 and 0200 hours) on metabolic markers and IGF1 level. They found that the same amount of rhGH given as the two large boluses generated similar and significant metabolic responses on glucose and lipid markers than at baseline. All three schedules resulted in a considerable increase in IGF1 levels, but the two IV bolus groups had lower peaks in IGF1 and quicker return to baseline than the other two schedules [10]. The authors speculated that the serum GH level might need to be sustained above zero for prolonged periods to achieve its growth-promoting effects. In our patient, daily IV rhGH boluses markedly improved linear growth despite a modest increase of serum IGF1 levels.

Growth hormone stimulates hepatic IGF1 production via the activation of transmembranous receptors. Whether the expression of liver GH receptors is altered in patients with protein C deficiency or the administration of protein C concentrate immediately before the rhGH boluses had suppressed the normal hepatic response is not known. There is no standard dose for IV rhGH replacement. Moreover, we did not assess this treatment's direct effects on lipid metabolism, body composition, or cardiovascular markers. These would have provided direct evidence on the metabolic benefits of rhGH treatment on this patient.

## 4. Conclusions

IV administration may be an alternative route for treating GHD in eligible patients with permanent vascular access if there are challenges or potential side effects of subcutaneous administration. A significant rise in serum IGF1 levels may not be needed to achieve the growth-promoting effect of IV rhGH therapy.

## Figures and Tables

**Table 1:** Laboratory results of selected biomarkers of the patient before and after growth hormone treatment.

Age (years, months)	2^5/12^ years (before rhGH)	3^2/12^ years (before rhGH)	5^2/12^ years (5 months after rhGH)	6^1/12^ years (16 months after rhGH)
IGF1 concentration (ng/mL)	<15	<15	29	41
IGF1 SDS score			−2.33	−2.17
IGFBP3 concentration (mg/L, RR: 0.8–3.9)	0.65	0.8	1.6	
TSH (mIU/L)	2.1		2.6	1.3
Free T4 (ng/dL)	1.1		0.9	0.9
Albumin (gr/dL)			4.3	
ALT (IU/L)			27	
AST (IU/L)			20	
HbA1c (%)			4.7	4.5
Protein C functional (%, RR: 40–92)	14	14	11	12

## Data Availability

Data are available on request from the corresponding author.
